# Characteristics of SARS-CoV-2 positive cases beyond health-care professionals or social and health-care facilities

**DOI:** 10.1186/s12889-020-10093-w

**Published:** 2021-01-07

**Authors:** Giovanna Deiana, Antonio Azara, Marco Dettori, Fiorenzo Delogu, Gavino Vargiu, Isabella Gessa, Antonella Arghittu, Marcello Tidore, Giorgio Steri, Paolo Castiglia

**Affiliations:** 1 Department of Medical, Surgical and Experimental Sciences, University of Sassari, Via Padre Manzella 4, 07100 Sassari, Italy; 2 Public Health Service, Local Health Unit, Sassari, Italy; 3 Department of Biomedical Sciences, University of Sassari, Sassari, Italy; 4 Assessorato dell’Igiene e Sanità e dell’Assistenza Sociale, Regione Autonoma della Sardegna, Sassari, Italy; 5 ATS Sardegna, Sassari, Italy

**Keywords:** SARS-CoV-2, COVID-19, Health-care professionals, Social and health-care facilities, Transmission dynamics, Italy

## Abstract

**Background:**

During the outbreak of SARS-CoV-2 in Italy, infection among health-care professionals and in the context of welfare and health-care facilities was a significant concern. It is known that the elderly or those with concomitant pathologies are at greater risk of a serious evolution of the disease if affected by COVID-19 and that health workers are a category with greater exposure to SARS-CoV-2 infection. Until now, there has been little information on the epidemiological features and transmission dynamics of the COVID-19 outbreak which did not involve health-care professionals or social and health-care facilities. For this reason, this paper aims to describe the epidemiological characteristics of SARS-CoV-2 infection in the general population outside these semi-closed communities.

**Methods:**

The study was designed by analyzing the data of the 1371 SARS-CoV-2 positive subjects observed in Sardinia up to 9 July, 2020 and whose data were available in the public health department. Statistical analysis and graphic representation were performed using STATA and Adobe Illustrator, respectively.

**Results:**

Of the positive cases analyzed, 323 (23.5%) are health-care workers and 563 (41.1%) reside in social or health-care facilities. The number of positive cases among the general population (subjects who do not belong to these semi-closed communities), is 399 (29.1%), 208 females and 191 males. The estimated Case Fatality Rate stands at 5.0%, which is almost half the rate reported for all the SARS-CoV-2 positive cases (9.8%). The geographical distribution of positive cases differs considerably from the distribution of the totality of cases in Sardinia.

**Conclusions:**

This review provides an insight into the COVID-19 situation in the general community, ie not involving health-care professionals or social and health-care facilities. Understanding the evolving epidemiology and transmission dynamics of the outbreak outside of these semi-closed communities would provide appropriate information to guide intervention policy. The COVID-19 pandemic has exacerbated the vulnerability of our health-care system. Severe disruptions in care, medicine shortages and unequal access to health-care are but a few examples of the challenges faced by people living in Italy and Europe, highlighting the importance of evidence-based approaches in supporting the development of prevention and response strategies for future pandemics.

## Background

The Coronavirus Disease 2019 (COVID-19), a clinical syndrome associated with Severe Acute Respiratory Syndrome Coronavirus 2 (SARS-CoV-2) infection, causes a respiratory syndrome with varying degrees of severity, ranging from asymptomatic or paucisymptomatic forms to severe interstitial pneumonia and acute respiratory distress syndrome (ARDS) which requires mechanical ventilation and support in an intensive care unit (ICU) [1, 2]. The frequency of asymptomatic infections is still uncertain. According to a narrative review, asymptomatic subjects appear to account for approximately 40 to 45% of SARS-CoV-2 infections while, based on the report of a recent seroprevalence survey by the Italian Ministry of Health, they account for 27.3% [3, 4].

COVID-19 is characterized by symptoms such as fever, dry cough, headache, asthenia, anosmia, ageusia and sore throat [5–7]. Given the 2–14-day incubation period of SARS-CoV-2, its high transmission potential and the similarity of its symptoms to those of the common cold, various studies have reported that most people ignored the infection, causing its increased transmission among individuals [8, 9].

Currently, as of August 7, 2020, according to a WHO report, COVID-19 has affected 18,725,088 patients and has caused a total of 703,389 deaths worldwide [10]. In particular, according to the Italian National Health Institute (*ISS - Istituto Superiore della Sanità*), Italy’s COVID-19 epidemic has assumed dramatic characteristics, showing 249,204 confirmed cases and 35,187 deaths [11] as of August 6, 2020, with a higher death toll than China and other European countries and with numerous differences in the various regional settings [12, 13]. Lombardy, in northern Italy, has a total of 96,952 confirmed cases and 16,833 deaths while the total for Basilicata, in the south, is 477 confirmed cases and 28 deaths [14].

During the outbreak, infection among health-care professionals and within residential care-homes and health-care facilities were a significant concern. In these structures, where people with disabilities, serious neurological or elderly diseases are in close contact with each other and with the staff (both health- and non-health-care) who assist them, the effects of the COVID-19 health emergency can be particularly serious.

It is known, in fact, that the elderly or those with concomitant pathologies are at greater risk of a serious evolution of the disease if affected by COVID-19 and that health workers are more exposed to SARS-CoV-2 infection than the general public [15, 16]. Furthermore, it is important to emphasize that these structures, as well as other semi-closed communities, are also at greater risk of epidemic micro-foci and as such they greatly contributed to the epidemiology of the disease.

On the other hand, so far, there has been little information on the epidemiological features and transmission dynamics of the COVID-19 outbreak that do not involve health-care professionals or health-care facilities. In fact, the epidemiological descriptions were first based on deaths for COVID-19 or hospital admissions, in particular those in intensive care units, and only subsequently did the study expand with seroepidemiological investigations on the general public. However, to estimate its real spread in the community it is important to describe the dynamics of contacts, aside from large outbreaks such as those occurring in hospitals, including among health personnel, and in nursing homes. For this reason, this paper aims to describe the epidemiological characteristics of SARS-CoV-2 infection in the general public, unrelated to these semi-closed communities. The study was conducted in Sardinia, an Italian region located in the center of the western Mediterranean Sea.

## Methods

### Study setting

The submission of the present study to our institutional ethics committee (Comitato Etico Indipendente della A.O.U. di Cagliari) was deemed unnecessary for its observational design according to the national regulation [17].

Sardinia, with a population of 1,639,591 inhabitants [18], lies in the center of the western Mediterranean Sea. Thanks to its geographical characteristics and in particular to its insularity, it has experienced phenomena such as isolation and little genetic flow and, thus, its population is relatively homogeneous and suitable for epidemiological studies [19]. Furthermore, being an island, as the epidemic spread in Italy it was quite easy for the Sardinian government to immediately close all borders avoiding any type of external interference.

The study was designed by analyzing the data of the 1371 SARS-CoV-2 positive subjects observed in Sardinia up to 9 July, 2020 and whose data were available in the public health department, part of these patients have been included in a previous study [12]. Information collected regarded age, gender, clinical status (synthetic indicator of the severity of the symptoms), the possible presence of several health conditions and potential risk factors (chronic underlying diseases) and the final outcome.

### Statistical and network analysis

Descriptive statistical analyses were expressed with absolute and relative (percentage) frequencies. CFR was calculated by comparing the number of SARS-CoV-2 deaths with the number of positive SARS-CoV-2 subjects. Statistical analysis was performed using STATA 16.1 (Stata Corp, College Station, TX, US).

The graphic representation of the spatial and temporal distribution of the positive cases under study was created by categorizing them by province and dividing them into a period of time dating from March 1st to July 7th subdivided by week. Different colors denote different genders. It was also indicated whether the infection was related to health-care workers or retirement homes or if the contagion was of unknown origin. The representation was performed using Adobe Illustrator CC 2019 (Adobe Inc., San Jose, CA, US).

## Results

**Fig. 1:**
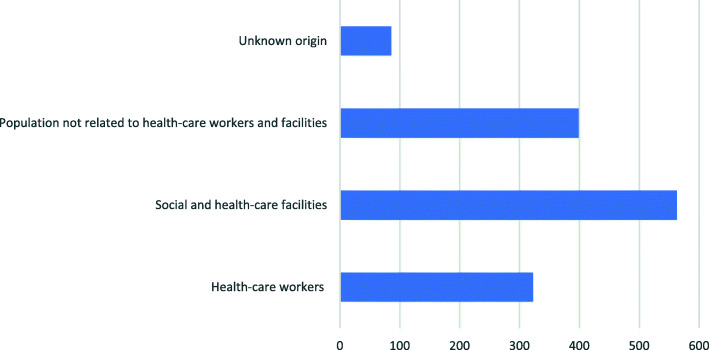
Distribution of SARS-CoV-2 positive cases in Sardinia

The number of SARS-CoV-2 positive cases was 1371 (814 females and 557 males, 59.4 and 40.6% respectively). Of the 1371 positive subjects, 399 cases (208 females and 191 males) occurred in the general public, i.e. were unrelated to health-care professionals or residential and health-care facilities, whereas 323 (23.5%) were health-care workers and 563 (41.1%) residing in residential and health-care facilities. No information on the origin of the infection were available for 86 cases (6.3%) (Fig. 1).

The mean age of the population under study was 51.0 +/− 20.6 years (median 52.2, range 0.6–94.6) which is lower than the mean age of patients who contracted the infection (59.4 +/− 20.7, median 57.3, range 0.6–99.4). Most case patients were in the age groups 50–59 and 40–49 (*n*= 86, 21.5% and *n*= 83, 20.8% respectively), 15.0% were under the age of 30 and 9.0% were 80 years of age or older. There was no difference in median age by sex (males: 52.6 years; females: 51.2 years) (Table 1).

**Table 1:** Distribution of diagnosed cases unrelated to health-care workers or residential homes and health-care facilities in Sardinia by age group and gender

Age groups	Females	% females	Males	% males	Total	% total
0–9	8	66.7	4	33.3	12	3.0
10–19	12	48.0	13	52.0	25	6.3
20–29	15	65.2	8	34.8	23	5.8
30–39	20	52.6	18	47.4	38	9.5
40–49	43	51.8	40	48.2	83	20.8
50–59	43	50.0	43	50.0	86	21.5
60–69	24	42.9	32	57.1	56	14.0
70–79	23	57.5	17	42.5	40	10.0
80–89	16	59.3	11	40.7	27	6.8
> 90	4	44.4	5	55.6	9	2.3
Total	**208**	**52.1**	**191**	**47.9**	**399**	**100**

At least one underlying condition or risk factor was present in 140 out of 353 (39.7%) patients for which data were available. The most commonly reported conditions were cardiovascular diseases (*n*=62, 44.3%) and chronic lung diseases (*n*=39, 27.9%). No information on underlying health conditions were available for 46 cases (11.5%). Of these 140 cases with underlying health conditions, 72 were female and 68 were male. As shown in Table 2, there were no differences between males and females for the presence of Diabetes Mellitus, cardiovascular diseases and chronic lung diseases. However, there was a higher prevalence of obesity and liver diseases in males and metabolic diseases and chronic neurological diseases in females.

**Table 2:** Underlying health conditions of the population under study

Underlying health condition	% females	% males	% total
Active tumors	33.3	66.7	6.4
Diabetes Mellitus	50.0	50.0	8.6
Cardiovascular diseases	48.4	51.6	44.3
HIV	66.7	33.3	4.3
Chronic lung diseases	51.3	48.7	27.9
Chronic renal disease	33.3	66.7	4.3
Metabolic diseases	80.0	20.0	10.7
Obesity	10.0	90.0	7.1
Liver diseases	0.0	100.0	3.6
Chronic neurological diseases	77.8	22.2	6.4

With 20 deaths, the estimated Case Fatality Rate (CFR) stands at 5.0%, which is almost half the rate reported for all the SARS-CoV-2 positive cases (9.8%). Of the patients who died, 35.0% were aged 80–89 years and 30.0% were aged 70–79 years (those aged > 90 years made up 10.0%). The number of deaths in patients over 70 years old accounts for 75.0% of the total. No deaths occurred in cases under the age of 40. Among the 20 deaths, 6 were female (30.0%) and 14 male (70.0%).

**Fig. 2:**
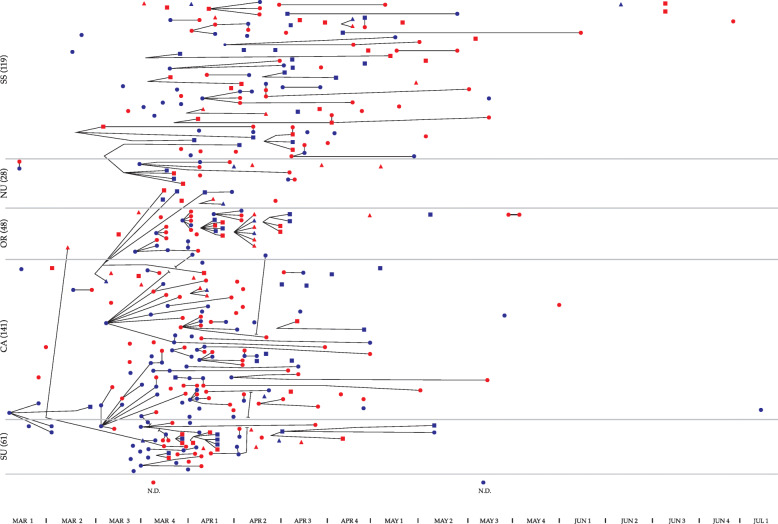
Spatial and temporal distribution of the positive cases under study in Sardinia. SS: Sassari, NU: Nuoro, OR: Oristano, CA: Cagliari, SU: Sud Sardegna, N.D.: for these particular cases the province has not been indicated. : the origin of the infection was attributable to health-care workers or social and health-care facilities, : the origin of the infection was unknown, : the origin of the infection was known but not attributable to health-care workers or social and health-care facilities. Red: females, Blue: males. MAR 1, MAR 2, MAR 3, MAR 4: first, second, third, fourth week of March

**Fig. 3:**
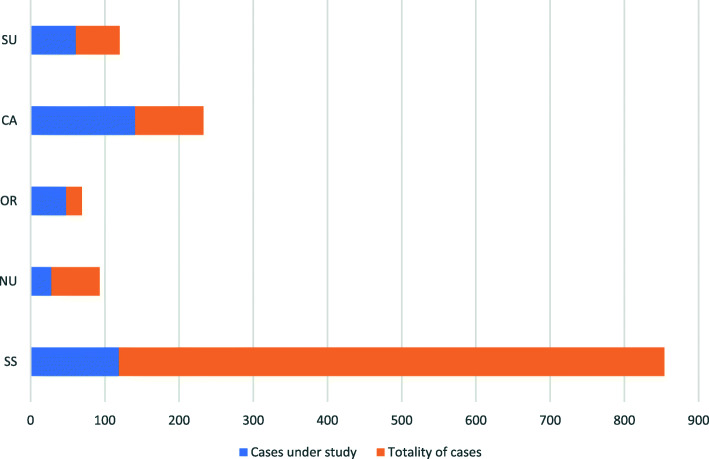
Cumulative cases of population under study and totality of cases in Sardinia divided by province. SS: Sassari, NU: Nuoro, OR: Oristano, CA: Cagliari, SU: Sud Sardegna

Among the cases analyzed, as shown in Fig. 2, 74 (18.5%) were apparently isolated cases, and infection occurred outside Sardinia in 32.4% The remaining cases (*n* = 325) formed clusters of various sizes with 29.2% attributable to health-care workers or residential and health-care facilities while, for 14.1% of cases, the origin of the infection was not identified. Most cases occurred in clusters of 2 (*n* =64, 19.7%) and 6 (*n* =60, 18.5%) subjects. Two large clusters of 13 and 14 cases stand out, linked to infections that originated in the workplace. From a geographical point of view, most of the cases under study were concentrated in the metropolitan city of Cagliari (*n*= 141, 35.3%), followed by the provinces of Sassari (*n*= 119, 29.8%), Sud Sardegna (*n*= 61, 15.3%), Oristano (*n*= 48, 12.0%) and Nuoro (*n*= 28, 7.0%). These data differ considerably from the geographical distribution of the totality of positive cases in Sardinia (1371) where we find 233 cases in Cagliari (17.0%), 854 in Sassari (62.3%), 120 in Sud Sardegna (8.7%), 69 in Oristano (5.0%) and 93 in Nuoro (6.8%). For 2 positive cases the province has not been indicated (Fig. 3).

**Fig. 4:**
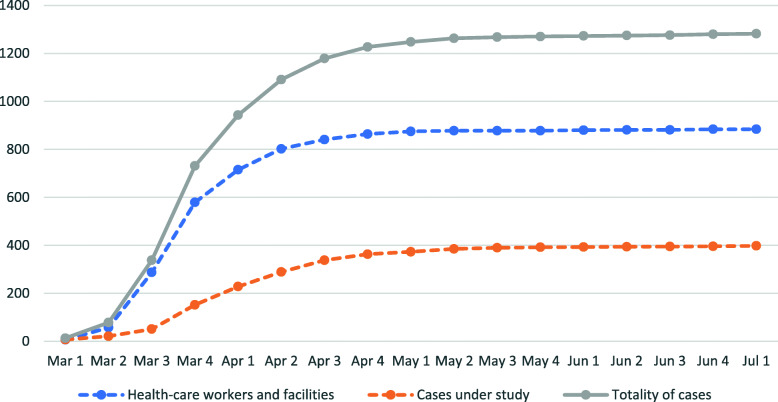
Cumulative trend over time of incident cases in Sardinia

Figure 4 describes a cumulative trend over time of incident cases split into health-care workers and facilities, cases under study and totality of cases in Sardinia.

## Discussion

The total number of people infected in Sardinia is 1371, two thirds of which (854) are concentrated in the province of Sassari and mostly in hospitals and retirement homes for the elderly. On the island, of 134 deaths from COVID-19, more than half occurred in retirement homes, prompting the prosecutors of Cagliari and Sassari to open an investigation. In our analysis, we observed transmission in a particular context, COVID-19 cases which did not involve health-care professionals or residential and health-care facilities and analyzed their epidemiological features and transmission dynamics.

Different clusters of various sizes were reported, a third of which involved health workers or nursing homes. Most of the remaining clusters were linked to familiar transmission, except for two large clusters with transmission within the workplace. Excluding health-care professionals and residential and health-care facilities there has not been a large-scale spread of the virus among the population.

These findings suggest that Italy has taken decisive action to tackle COVID-19 transmission. Infection control measures taken include border control, isolation of cases, quarantine of contacts, mandating mask-wearing in certain situations, promoting hand hygiene and placing strict restrictions on personal movement, as well as resource re-allocation, reassurance and education of the public and broadcasting a daily government press conference [20].

Sardinia, in particular, immediately closed all borders as cases rose throughout Italy and, being an island, it was quite straightforward to shut down all airports and maritime access points, thus avoiding any type of external interference except for the essential supplies of food, sanitary material, raw materials and consumer goods. As a consequence, the low number of infections may have protected the general public, ie those who are not health-care professionals nor residents in care-homes and health-care facilities.

However, it is important to underline the presence of certain differences in the distribution of cases from a geographical point of view. In the southern part of Sardinia, in fact, there was a greater tendency for the formation of clusters, even small ones, while in the north the contagion was from person to person and more delayed over time. This probably highlights, in the latter case, a greater effectiveness of the territorial public health in controlling cases and their contacts.

As a matter of fact, as the epidemic progressed, SARS-CoV-2 positive cases were isolated more quickly and their contacts identified and quarantined, ensuring that further cases were rapidly detected and isolated when they developed symptoms. We can therefore affirm that strict containment measures and the increased awareness of the population may have successfully contributed to containing the local transmission of SARS-CoV-2 within Italy [21].

The COVID-19 pandemic has exacerbated and exposed the vulnerability of our health-care system. Severe disruptions to care, medicine shortages and unequal access to health-care are but a few examples of the challenges faced by people living in Italy and Europe as a whole, highlighting the importance of evidence-based approaches and health literacy in supporting the development of prevention and response strategies for future pandemics [22].

To monitor the situation and give support to health-care workers, the Italian National Institute of Health (*ISS - Istituto Superiore di Sanità*) is engaged on several fronts with surveillance activities aimed at identifying any strategies for strengthening the infection control prevention programs (Infection prevention and control, IPC) and with support activities aimed at providing resources and indications in the areas of prevention and preparation the management of any suspected or confirmed cases of COVID-19 [23].

This study presents certain limitations. First of all, the typical limitations concerning the analysis of a rapidly-evolving infectious disease epidemic, including biases related to case ascertainment and non-homogeneous sampling over time. Secondly, data are missing for asymptomatic cases, consequently the real prevalence of COVID-19, its spectrum of presentation and the real lethality rate remain unknown. Moreover, given the small sample size, all of the results presented should be interpreted with caution. On the other hand, to the best of our knowledge, this is one of the first studies to focus on the epidemiological features and transmission dynamics of the COVID-19 outbreak in the general public, not including health-care professionals and residential and health-care facilities. Moreover, all data collected, coming from the public health department, are very reliable and the statistical analysis of our study was strong in its consistency.

## Conclusions

This review provides an insight into the COVID-19 situation in the general public, excluding health-care professionals and care-homes and health-care facilities. Understanding the evolving epidemiology and transmission dynamics of the outbreak outside of these semi-closed communities would provide appropriate information to guide intervention policy. Prevention is, at this point, the best practice in order to reduce the impact of COVID-19, also taking into account the fact that that there is currently no vaccine available [24, 25].

Furthermore, while in Sardinia community transmission of COVID-19 did not manifest itself in a particularly violent form, it is our opinion that it could have been further reduced through certain improvements such as reinforcing the role and increasing the staff of the public health department, better management of patients at home and greater availability of personal protective equipment, swabs and laboratories authorized to process samples.

Overall, much about COVID-19 still remains unknown and continuous monitoring of affected patients is still necessary, especially with regard to lethality and its capacity to spread on an epidemic level. In conclusion, only once the pandemic ends, will researchers be able to assess its health, social and economic impact and, hopefully, be prepared, especially with regard to public and global health, for any future similar pandemics.

## Data Availability

The datasets used and/or analyzed during the current study are available from the corresponding author on reasonable request. The data was not publically available, we obtained permission to access the data from the Public Health Service and from Assessorato dell’Igiene e Sanità e dell’Assistenza Sociale, for research purposes. Some of these data were used for a previous study.

## Abbreviations

COVID-19: Coronavirus Disease 2019
SARS-CoV-2: Severe Acute Respiratory Syndrome Coronavirus 2
ARDS: Acute Respiratory Distress Syndrome
ICU: Intensive Care Unit
ISS: Istituto Superiore della Sanità - Italian Higher Health Institute
CFR: Case Fatality Rate
IPC: Infection prevention and Control
